# Stapled Anastomosis for Side-to-Side Cavo-Cavostomy in Orthotopic Liver Transplantation

**DOI:** 10.3390/jcm12165289

**Published:** 2023-08-14

**Authors:** Emilia Kruk, Piotr Kalinowski, Krzysztof Gibiński, Krzysztof Dudek, Michał Skalski, Marta Przybysz, Andriy Zhylko, Łukasz Nazarewski, Marcin Morawski, Michał Grąt

**Affiliations:** 1 Department of General, Transplant and Liver Surgery, Medical University of Warsaw, 02-091 Warsaw, Poland; emiliakruk1@gmail.com (E.K.); michal.grat@wum.edu.pl (M.G.); 2 2nd Department of Clinical Radiology, Medical University of Warsaw, 02-091 Warsaw, Poland

**Keywords:** liver transplantation, inferior vena cava anastomosis, piggy-back anastomosis, stapled vascular anastomosis, stapled vena cava anastomosis, stapled piggy-back anastomosis, vascular stapler

## Abstract

In liver transplantation, a side-to-side anastomosis is one of the commonly performed techniques of the inferior vena cava reconstruction. The authors report a case of an application of an endoscopic vascular linear stapler for a side-to-side caval anastomosis during deceased-donor liver transplantation. The back table procedure was performed in a standard fashion for a side-to-side anastomosis. The linear vascular stapler was introduced during the temporary clamping of the recipient’s inferior vena cava and the anastomosis was created without problems. Suturing of the resulting defect completed the anastomosis. The use of the stapler resulted in a shortening of the anastomosis time. The staple line after the reperfusion of the graft was completely sealed. The patient’s postoperative course was uncomplicated and post-operative ultrasound and computed tomography confirmed the patency of the anastomosis. This case demonstrates a novel approach to a side-to-side caval reconstruction during liver transplantation that enables a shortening of the implantation time and may improve the quality of anastomoses.

## 1. Introduction

Since the first orthotopic liver transplantation was performed in 1963 by Starzl et al. [1] the operative technique has gone through modifications to reduce the ischemia time, avoid hemodynamic complications induced by caval clamping and minimize the risk of vascular complications, including venous outflow obstruction. Improvements to the classical technique of end-to-end cavo-caval anastomosis include the introduction of a piggy-back technique originally described in 1968 by Calne et al. [2] that preserves the recipient’s inferior vena cava (IVC), reducing hemodynamic impairment during liver transplantation and shortening the total operative time. A modification of the piggy-back technique was presented by Belghiti et al. in 1992 and is now commonly used in many liver transplantation centers [3,4]. According to a recently published review and expert opinion, none of the available techniques can be recommended over the others [5]. The wide availability of novel mechanical devices allows for improvements in surgical technique and a further reduction in the time of cavo-caval anastomosis during liver transplantation. One of the approaches is the use of a linear stapler for a modified piggy-back cavo-cavostomy, but experience with this technique is limited to incidental reports. The first report of this technique was published in 2009 by Quintini et al., who described a side-to-side (modified piggy-back) stapled anastomosis technique as a rescue procedure for severe hepatic venous outflow obstruction after a standard piggy-back anastomosis [6]. The first report of a successful elective stapled piggy-back anastomosis in a human was published in 2013 by Akbulut et al. [7], but the technique has not been further developed or investigated in randomized studies. Meanwhile, linear vascular staplers have gained acceptance and are routinely used in major liver resections for closure and division of the main hepatic veins, portal pedicles, and transection of hepatic parenchyma [8,9,10]. Linear vascular staplers have also been used for elongation of the right renal graft vein for transplantation [11,12]. In this article, we would like to present our initial experience with linear stapled side-to-side cavo-caval anastomosis with the restoration of the IVC blood flow for adult orthotopic liver transplantation. The operative technique is presented and the current literature on the subject is reviewed with a discussion of the pros and cons of this novel approach.

## 2. Case Report

A 43-year-old male with hepatitis C virus (HCV)-related liver cirrhosis underwent elective deceased-donor orthotopic liver transplantation at the Department of General, Transplant and Liver Surgery of the Medical University of Warsaw. At the time of transplantation, his Model for End-Stage Liver Disease (MELD) and Child–Pugh scores were 19 and 9, respectively. A hepatectomy with preservation of the recipient’s IVC was performed. The whole-liver graft procurement from the brain-dead donor and preparation for piggy-back transplantation was performed as described previously [13]. The liver graft weighed 2000 grams and was anatomically compatible with the recipient, which resulted in a tight fit in the subdiaphragmatic space, but inferior exposure during caval anastomosis was expected. The stapled anastomosis was chosen as a method to facilitate safe anastomosis in a limited space. The superior and inferior orifices of the graft’s IVC were closed using 4-0 polypropylene monofilament running sutures. Tourniquet tapes were placed under IVC, and two straight vascular clamps were applied at the suprahepatic and infrahepatic IVC, interrupting completely the venous return through the IVC.

**Figure 1:**
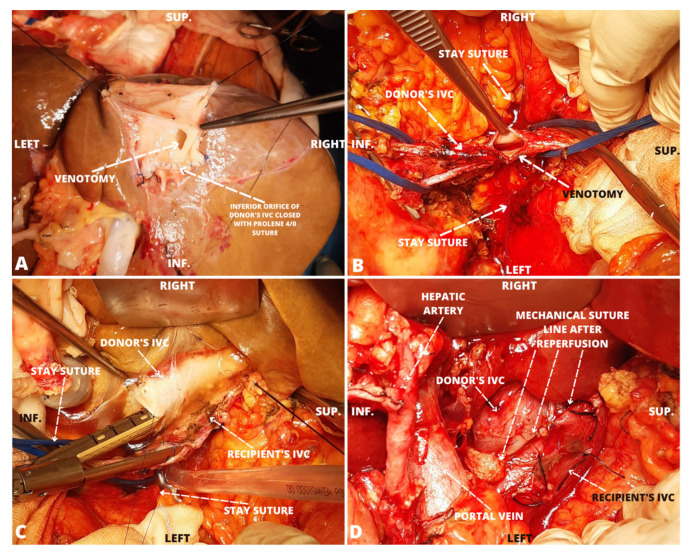
A side-to-side linear stapled anastomosis of the inferior venae cavae during orthotopic liver transplantation. Preparation of IVCs of the graft (**A**) and recipient (**B**) with corresponding venotomies. Placement of the stapler (**C**). The vascular anastomoses after a reperfusion (**D**). IVC—the inferior vena cava, INF.— inferior, SUP.—superior.

A one-centimeter incision was made in the distal part of the recipient’s IVC and a corresponding venotomy was performed in the IVC of the liver graft (Figure 1A,B). Two stay sutures were applied to aid the proper alignment of the IVCs of the graft and the recipient. After placing the graft in the abdominal cavity, the jaws of the endoscopic stapler (EndoGIA 45 mm vascular reload) were inserted into the lumens of both IVC segments through prepared venotomies. The orientation of the stapler reload was parallel to the axis of the IVC (Figure 1C). The staples were fired, creating a side-to-side cavo-cavostomy. The suture line was inspected and appeared properly sealed. The time needed to complete the anastomosis including preparation and cross-clamping was less than 10 min. Throughout the process of execution of the cavo-cavostomy, the graft was flushed with cold saline to remove the preservation solution and keep the graft cooled. In the control laboratory test of perfusate, the level of potassium was 11.4 mmol/L; therefore, closure of venotomy in the IVCs was postponed and graft flushing continued. A Satinsky–DeBakey clamp was placed on the IVC of the recipient posteriorly to the staple line and the straight vascular clamps were removed, thus allowing for the partial restoration of blood flow in the recipient’s IVC. The patient tolerated the short period of cross-clamping well. The portal and arterial anastomoses were performed in a standard fashion using two running polypropylene monofilament 5-0 and 6-0 sutures, respectively. In the second test, the potassium concentration in the flushing solution was 8.4 mmol/L. The stay sutures were removed and the incisions in the IVCs were closed transversely with a 4-0 polypropylene monofilament continuous suture. The resulting anastomotic line length was 55 mm, consisting of a 45 mm staple line and a 10 mm sutured venotomy closure. Reperfusion was performed after 8 h and 45 min of cold ischemia time. The warm ischemia time was 38 min. No areas of hypoperfusion or blood flow congestion in the graft were apparent immediately after the reperfusion, and there was no bleeding from the stapled IVC anastomosis (Figure 1D).

**Figure 2:**
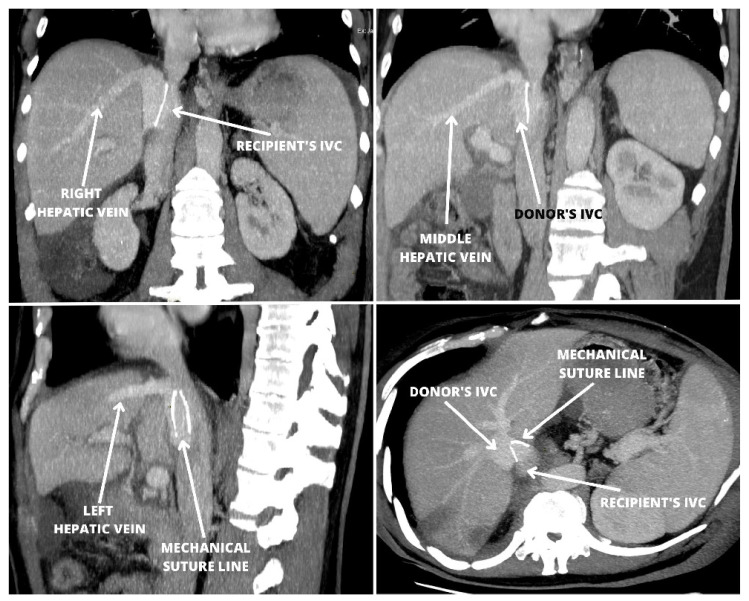
A follow-up computed tomography performed on the 12th postoperative day in the patient with stapled cavo-caval side-to-side anastomosis showed patent vascular anastomoses including anastomosis of the IVCs with no signs of hepatic venous obstruction. IVC—inferior vena cava.

No post-reperfusion syndrome was observed. The biliary ducts were anastomosed end-to-end with two running 6-0 polydioxanone sutures. The postoperative period was uneventful. An ultrasound examination on postoperative day (POD) one and POD 5 showed patent vascular anastomoses with typical flow characteristics. There was no stricture observed in the cavo-caval anastomosis and the maximal blood flow velocity (Vmax) equaled 73 cm/s. On POD 7, the patient’s laboratory results revealed normalization of the liver function (Table 1). Follow-up computed tomography performed on POD 12 revealed patent vascular anastomoses including piggy-back anastomosis of the IVCs with no hepatic venous obstruction (Figure 2). The patient was discharged on POD 12 in good clinical condition. On follow-up examinations on POD 30 and POD 60, the patient remained well with good graft function and laboratory results within the normal range (Table 1). Recently, another two patients were transplanted using the same stapled IVC anastomosis technique, with an uneventful immediate postoperative course and excellent outflow hemodynamics.

## 3. Discussion

**Figure 3:**
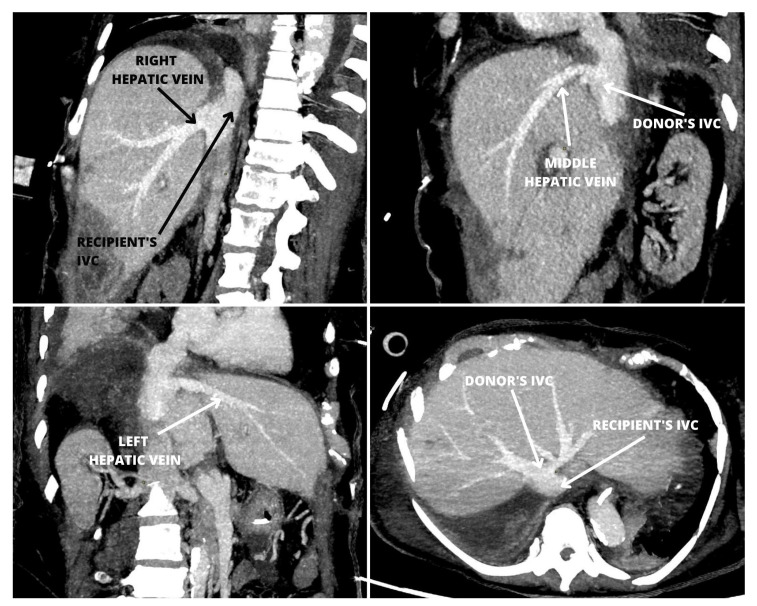
Computed tomography of a patient after liver transplantation with a sutured side-to-side inferior venae cavae anastomosis shows an anatomy similar to a stapled anastomosis.

A modified cavo-caval side-to-side anastomosis with a linear stapler and partial restoration of the IVC blood flow during orthotopic liver transplantation was presented. The authors chose the simplest possible technique of performing a stapled side-to-side cavo-cavostomy with a single linear cartridge application and closure of the resulting defect with a continuous suture. The authors believe this type of anastomosis most closely mimics the sutured piggy-back anastomosis and offers at least the same quality and safety of the resulting reconstruction (Figure 2 and Figure 3). The implantation time is a factor influencing the outcome of liver transplantation [14]. Utilization of the stapled anastomosis technique results in a shortening of the period of cavo-caval anastomosis, which contributes significantly to the time of all anastomoses. An additional modification of the implantation technique, which was a consequence of a short time of caval anastomosis, was the creation of the arterial anastomosis before portal anastomosis to continue cold flushing of the graft. Recently, Sutherasan et al. compared three commonly applied techniques for IVC anastomosis and reported the shortest warm ischemia time in patients with side-to-side cavo-caval anastomoses, which averaged 40 min [15]. It may be further reduced with the stapled technique.

The piggy-back technique reduces the risk of acute kidney injury (AKI) after transplantation compared to the caval-replacement technique due to the preservation of the IVC blood flow [16]. This benefit of sutured piggy-backing may be affected during stapled side-to-side anastomosis due to a need for temporary cross-clamping of the recipient IVC, which contributes to venous stasis and a decreased cardiac output. A possible solution to diminish the effects of cross-clamping in a patient with poor tolerance is conversion to longitudinal clamping of the IVC with a Satinsky clamp and partial restoration of the IVC blood flow immediately after firing of the linear stapler. This maneuver together with a shorter warm ischemia time may outweigh the risk factors of AKI. In the presented case, the patient tolerated the period of interrupted venous return well and no signs of kidney injury were observed.

Liu et al. in 2006 published an experimental study on an EndoGIA linear stapler for IVC anastomosis in dogs [17]. The allograft segment of the IVC was left open at both ends and after the linear stapled side-to-side anastomosis the proximal and distal openings were closed in a U-shaped fashion. This additional maneuver was intended to prevent the creation of a dead space inside the IVC allograft. The influence of the anastomotic technique on the risk of outflow obstruction in orthotopic liver transplantation is a matter of ongoing dispute. Sutherasan et al. found no difference in the hepatic venous outflow complication rate among the three techniques of IVC anastomosis [15]. Nevertheless, venous outflow obstruction is uncommon but a serious complication observed in 0.5–3.0% of patients after a full-graft classic piggy-back implantation [18,19,20] Various preventive solutions were described, including side-to-side anastomosis (modified piggy-back) and a range of venoplasties, such as a widening of the ostium of the recipient’s hepatic veins [21]. Therapeutic options include medical therapy in mild cases, balloon angioplasty with or without placement of a stent in the narrowed hepatic vein, surgical venoplasty, reconstruction of the IVC anastomosis or retransplantation [19,22,23]. This problem may be also managed with a rescue stapled side-to-side anastomosis of the IVCs as recently described by Quintini et al. [6].

Stapled IVC anastomosis is a useful technique in voluminous liver grafts or cases of large caudate lobes when access to the proximal part of the IVC may be cumbersome. The placement of an EndoGIA stapler and suturing of the defect in the IVCs is performed from the caudal approach, which requires only minimal retraction of the liver to expose the infrahepatic IVCs. Moreover, sutured anastomoses in bulky grafts may be prone to excessive tension on the suture line, resulting in minimal lacerations in the vessel wall. This frequently leads to oozing from the anastomotic line after the restoration of blood flow through the liver and the IVC. Another possible problem with sutured anastomosis under tension may be its shortening and an increased risk of outflow obstruction. However, it has to be noted that the limitations of sutured modified piggy-back anastomosis also pertain to the stapled piggy-back technique. Anatomic limitations, abnormalities of the IVC, or severe adhesions between the liver and the retrohepatic IVC preclude safe preservation of the IVC during hepatectomy [24]. Voluminous liver grafts in a limited space may be better managed with a classical implantation technique with replacement of the retrohepatic IVC to obviate the risk of excessive compression on a side-to-side anastomosis.

## 4. Conclusions

The stapled side-to-side cavo-caval anastomosis during orthotopic liver transplantation may offer some benefits, including shortening of the graft ischemia period, improved feasibility of anastomosis in large liver grafts or inconvenient caudate anatomy and better quality of the anastomotic line. However, some disadvantages and risks are possible, including less control of the anastomotic line, the lack of a way to adjust during the placement of staples, the risk of leaving loose staples in the venous lumen and the possible increased rigidity at the apex of the anastomosis. Well-designed randomized studies are needed to assess the clinical significance of the expected advantages and disadvantages of such an approach.

## 5. Future Directions

Stapled cavo-caval anastomosis is a promising modification of the piggy-back liver transplantation technique. Other devices for vascular anastomosis may become future alternatives, including non-penetrating single staples [25] or even tissue adhesives recently evaluated for vascular anastomoses in a porcine model [26].

## Figures and Tables

**Table 1:** The patient’s laboratory results preoperatively and 7, 30 and 60 days of follow-up after liver transplantation with stapled IVC anastomosis.

	At Admission	POD 7	POD 30	POD 60
ALP (IU/L)	69	325	147	87
GGTP (IU/L)	23	310	107	36
AST (IU/L)	18	37	27	20
ALT (IU/L)	18	151	52	29
Tot. Bil (mg/dL)	2.72	1.78	0.86	0.53
INR	1.10	1.13	1.11	1.11
Albumin (g/dL)	3.4	3.4	4.5	4.4
PLT (103/μL)	167	246	237	164
WBC (103/μL)	5.34	7.82	4.76	3.29
Hgb (g/dL)	9.3	8.8	9.9	9.9
Creatinine (mg/dL)	0.98	0.59	0.89	1.06

POD—postoperative day; ALP—alkaline phosphatase; GGTP—gamma glutamyl transpeptidase; AST—aspartate transaminase; ALT—alanine transferase; Tot. Bil.—total serum bilirubin; INR—international normalized ratio; PLT—platelet count; WBC—white blood cell count; Hgb—hemoglobin.

## Data Availability

Data are contained within the article.

## Abbreviations

The following abbreviations are used in this manuscript:

AKI	acute kidney injury
ALP	alkaline phosphatase
ALT	alanine transferase
AST	aspartate transaminase
GGTP	gamma glutamyl transpeptidase
HCV	hepatitis C virus
Hgb	hemoglobin
INF.	inferior
INR	international normalized ratio
IVC	inferior vena cava
MELD	Model for End-Stage Liver Disease
PLT	platelet count
POD	postoperative day
SUP.	superior
Tot. Bil.	total serum bilirubin
Vmax	maximal blood flow velocity
WBC	white blood cell count
